# Insulin‐like growth factor 1 ameliorates pre‐eclampsia by inhibiting zinc finger E‐box binding homeobox 1 by up‐regulation of microRNA‐183

**DOI:** 10.1111/jcmm.17403

**Published:** 2023-03-29

**Authors:** Weisi Lai, Ling Yu

**Affiliations:** ^1^ Department of Obstetrics and Gynecology, Second Xiangya Hospital Central South University Changsha China

**Keywords:** angiogenesis, insulin‐like growth factor 1, invasion, microRNA‐183, preeclampsia, proliferation, zinc finger E‐box binding homeobox 1

## Abstract

As a common hypertensive complication of pregnancy, preeclampsia (PE) remains one of the leading causes of maternal and fetal with high morbidity and mortality worldwide. Much research has identified the vital functions of insulin‐like growth factor 1 (IGF‐1) in PE treatment. However, the combined roles and molecular mechanism of IGF‐1 and microRNAs (miRNAs) underlying PE remain unclear. Therefore, we first measured the expression of IGF‐1, zinc finger E‐box binding homeobox 1 (ZEB1) and microRNA‐183 (miR‐183) expression in the placenta tissues of patients with PE by Western blot analysis and RT‐qPCR. Interactions among IGF‐1, ZEB1 and miR‐183 were assessed by Western blot analysis, ChIP‐PCR and dual‐luciferase reporter gene assay. The effect of IGF‐1 on the biological characteristics of trophoblast cells was investigated by CCK‐8, colony formation assay and in vitro angiogenesis experiments after cells were transfected with si‐IGF‐1. Finally, a mouse eclampsia model induced by knockdown of IGF‐1 was established to confirm the in vitro effect of IGF‐1 on PE. We found that IGF‐1, ZEB1 and miR‐183 were highly expressed in the placental tissues of patients with PE. The knockdown of IGF‐1 resulted in reduced proliferation and invasion of trophoblast cells and was accompanied by inhibited angiogenesis. ZEB1 was positively regulated by IGF‐1 via ERK/MAPK pathway, which in turn inhibited miR‐153 expression by binding to the miR‐183 promoter. The in vitro experiments further confirmed that IGF‐1 knockdown could induce PE. To sum up, IGF‐1 knockdown elevated expression of miR‐183 by downregulating ZEB1, thereby promoting deterioration of PE.

## 1 INTRODUCTION

Preeclampsia (PE) is a heterogeneous and common human‐specific pregnancy complication related to impaired placental development, which can jeopardize maternal and foetal lives if proper measures are not taken.^1^, ^2^, ^3^ The prevalence of PE has been reported to be 3%–5%.^4^ Delayed maternal age, overweight and vascular diseases have been implicated as important risk factors for PE.^5^ The current clinical management strategies available for PE are delivery of the placenta or preterm‐fetus.^6^, ^7^ However, present understandings of the intricate relationships among respective PE disease pathways are limited, which hinders us from developing better methods for successful management and prevention of PE.^8^ Therefore, this study was designed to explore a promising new therapeutic target to alleviate PE at the molecular level.

As a mitogenic hormone, insulin‐like growth factor 1 (IGF‐1) has been indicated to be closely related to various biological processes such as growth, metabolism, angiogenesis and differentiation. ^9^ Previous research has confirmed that IGF1 was involved in the compensation for placental deficits by enhancing the proliferation of smooth chorion extravillous cytotrophoblasts.^10^ Besides, another recent study has demonstrated that IGFs are important biomarkers in the prediction of PE.^11^ Moreover, IGF1 has been reported to be associated with PE by mediating the expression of storkhead box 1 in extravillous trophoblasts.^12^ Zinc finger E‐box binding homeobox 1 (ZEB1) is a molecule that is predominantly involved in the epithelial‐to‐mesenchymal transition (EMT) of various cancers.^13^ Nevertheless, ZEB1 has been found to be a crucial regulator in the progression of placental maturation.^14^ In addition, it has also been confirmed that IGF‐1 can repress the EMT in melanoma‐initiating cells and thus affect the downstream expression of ZEB1.^15^ MicroRNAs (miRNAs) are a group of small non‐coding RNAs that have been reported have an association with the pathogenesis of many diseases, including cancers, gestational diabetes and placental miRNAs dysregulation, which can induce pregnancy complications.^16^, ^17^, ^18^ Besides, miRNAs have also been identified as the vital regulatory molecules participating in the progression of PE.^19^ For instance, miR‐126 has been identified as a potential biomarker for PE pathogenesis.^20^ Furthermore, miR‐558 has been indicated to exert a promoting effect on the invasion and migration of trophoblasts, thereby participating in the progression of PE.^21^ More importantly, miR‐183 has also been confirmed to be a serum biomarker for PE.^22^ Meanwhile, a previous report has illustrated that miR‐183 can be inhibited by ZEB1.^23^ Based on the above‐mentioned literature, we speculated that IGF1 could mediate miR‐183 expression by regulating ZEB1 expression. However, it is still largely uncertain whether the same effects will be exerted in the context of PE. Hence, the present study was conducted to explore the regulatory role of IGF1 in the molecular mechanism underlying PE and ultimately provide a novel therapeutic target for the treatment of PE.

## 2 MATERIALS AND METHODS

### 2.1 ETHICS STATEMENT

This study was approved by the Obstetrics Ethics Committee of Second Xiangya Hospital, Central South University, and written informed consent was obtained from each participant. Human researches were conducted in strict accordance with the Declaration of Helsinki. All animal experiments were performed under the approval of the Animal Ethics Committee of Second Xiangya Hospital, Central South University.

### 2.2 Tissue sample collection

36 female participants including 18 patients with PE and 18 healthy women in late pregnancy were recruited as study subjects to donate placenta tissue samples. Patients with PE were diagnosed according to guidelines recommended by the American College of Obstetricians and Gynaecologists. Placental tissues that were related to severe maternal complications and fetal malformations were excluded from this study. Placental tissue samples (about 0.5 × 0.5 cm) were separated from the lateral central part of the maternal placenta as soon as possible after delivery. The collected tissues were washed with sterile phosphate‐buffered saline (PBS) to remove blood cells. Portions of the samples were fixed with 4% paraformaldehyde for 24–48 h and paraffin‐embedded for immunohistochemistry (IHC), whereas the remaining tissues samples were immediately frozen in liquid nitrogen immediately and stored at −80°C for the subsequent extraction of protein, RNA and DNA. HTR‐8/SVneo cells were purchased from american type culture collection (ATCC) and incubated in 1640 medium containing 10% foetal bovine serum (FBS, GIBCO, Invitrogen,), 1% penicillin and streptomycin (Invitrogen) at 37°C with 5% CO_2_.

### 2.3 Immunohistochemistry (IHC)

Paraffin sections were dewaxed with xylene conventionally, hydrated with gradient alcohol (anhydrous ethanol, 95% ethanol and 75% ethanol for 3 min each), treated in citrate repair solution and blocked with TBS solution containing 10% normal serum and 1% bovine serum albumin (BSA) for 2 h. Then, the sections were added with normal serum of rabbit polyclonal antibody to insulin‐like growth factor 1 (IGF‐1, ab9572, 1: 1000, Abcam,) dropwise as negative control (NC) and incubated in a refrigerator at 4°C overnight. Next, 50 μl of 3% H_2_O_2_ were added into each section and incubated for 20 min to eliminate endogenous peroxidase activity. In addition 50 μl of polymer enhancer and 50 μl of goat anti‐rabbit secondary antibody (ab205718, 1: 2000; Abcam) were added into section and incubated for 30 min at 37°C, respectively. Finally, each section was added with 100 μl freshly prepared diaminobenzidine (DAB) developing solution and observed under the microscope for 3–10 min, with positive tissues presenting as brown colour. Then, the sections were rinsed with distilled water, counterstained with haematoxylin, dehydrated with gradient alcohol (75% ethanol, 95% ethanol and anhydrous ethanol), sealed with neutral resins and observed again under the microscope.

### 2.4 Western blot analysis

Total proteins in tissues or cells were extracted using radioimmunoprecipitation assay (RIPA) lysis buffer containing phenylmethylsulfonyl fluoride (PMSF). The total protein concentration was measured using the BCA kit (P0012, Beyotime Biotechnology Co., Ltd.,). Then, 50 μg of proteins were dissolved in 2 × sodium dodecyl sulphate (SDS) loading buffer after boiled for 10 min, and then subjected to SDS‐polyacrylamide gel electrophoresis (PAGE) gel electrophoresis. The protein samples were then transferred onto the polyvinylidene fluoride (PVDF) membrane by the wet transfer method. The protein samples were then incubated with primary antibodies: rabbit anti‐IGF‐1 (1: 1000, ab9572, Abcam), rabbit anti‐ zinc finger E‐box binding homeobox 1 (ZEB1, 1: 1000, ab245283, Abcam), rabbit anti‐extracellular signal‐regulated kinase 1/2 (ERK1/2, 1: 1000, ab17942, Abcam), rabbit anti‐phosphorylated‐ERK1/2 (1: 1000, ab201015, Abcam), murine anti‐glyceraldehyde phosphate dehydrogenase (GAPDH, 1: 10000, ab8245, Abcam) at 4°C overnight. The protein samples were then incubated with secondary antibody immunoglobulin G (IgG, ab205718, goat anti‐rabbit, 1: 20000; ab205719, goat anti‐mouse, 1: 20000, Abcam) for 1 h and washed with TBST. Development was carried out with the use of sensitized chemiluminescent substrate electrochemiluminescence (ECL) (WBKLS0100, Millipore, Billerica, MA, USA). The grey value corresponding to protein expression was determined by Image J software.

### 2.5 Cell transfection

HTR‐8/SVneo cells were transfected with plasmids containing transcripts of si‐IGF‐1, overexpressed (oe)‐IGF‐1, si‐ZEB1 or oe‐ZEB1, respectively (plasmids were all purchased from GenePharma,). Transfection was conducted according to the manufacturer's instructions of Lipofectamine™ 2000 Transfection Reagent (11,668,019, Invitrogen), and their expressions were assessed by performing RT‐qPCR or Western blot analysis after 48 h of transfection.

### 2.6 Dual‐luciferase reporter gene assay

The promoter sequence and the full sequence of miR‐183 were obtained from the National Center of Biotechnology Information database (http://www.ncbi.nlm.nih.gov/gene). The promoter region of miR‐183 was cloned into the pmiRGLO (Promega,) luciferase vector to construct a pmiRGLO‐miR‐183 prom wild type vector (i.e., miR‐183 prom wt vector). HTR‐8/SVneo cells were transfected with si‐ZEB1 or oe‐ZEB1 according to the manufacturer's instructions of Lipofectamine™ 2000 and transfected with an empty vector as the control group. The miR‐183 prom wt vector and Renilla luciferase expression vector pRL‐TK (TaKaRa,) were transfected as internal references. After 24 h of transfection, a dual‐luciferase activity assay was performed according to the manufacturer's instructions of the Dual‐Luciferase Reporter Assay System (Promega).

### 2.7 Transwell assay

HTR‐8/SVneo cells invasion ability was detected using a Transwell filter (8 μm; Corning Incorporated,). The apical chamber of the basement membrane was coated with 50 μl of matrix gel (BD Biosciences, ). HTR8/SVneo cells were placed in the apical chamber of the serum‐free medium after transfected with controls or siRNA, whereas 20% FBS was placed in the basolateral chamber of the medium. Then, cells were cultured for 48 h with 5% CO_2_ at 37°C to detect the invasion ability of the cells. HTR‐8/SVneo cells that invaded to the basolateral chamber were fixed with 4% paraformaldehyde and stained with crystal violet. The total number of invaded cells was counted in five randomly selected fields.

### 2.8 RNA isolation and quantitation

The total RNAs in tissues or cells were extracted in strict accordance to the manufacturer's instructions of the TRIzol kit (cat: 15596018; Invitrogen), whereas the RNA concentration was determined using RT‐qPCR. The cDNA was synthesized using the ImProm‐II™ reverse transcription system kit. The reverse‐transcribed cDNA was diluted to 50 ng/μl for subsequent fluorescent quantitative PCR. The reaction amplification system was employed according to the manufacturer's protocols of Synergy Brands (SYBR) premixed ExTaq II kit (Takara). β‐actin was taken as an internal reference. The primer sequences used for RT‐qPCR are listed in Table 1.

**TABLE 1:** Primer sequence for RT‐qPCR

Genes	Primer sequence (5′‐3′)
IGF‐1	Forward: TCCTCGCATCTCTTCTACCT
	Reverse: AAAAGCCCCTGTCTCCACAC
ZEB1	Forward: CTCTGATTCTACACCGC
	Reverse: TGTCACATTGATAGGGCTT
miR‐183	Forward: ACACTCCAGCTGGGTATGGCACTGGTAGAA
	Reverse: CTCAACTGGTGTCGTGGAGTCGGCAATTCAGTTGAGA
β‐actin	Forward: GGCACCACACCTTCTACAATG
	Reverse: GGGGTGTTGAAGGTCTCAAAC
U6	Forward: CTCGCTTCGGCAGCACA
	Reverse: AACGCTTCACGAATTTGCGT

Note: IGF‐1, insulin‐like growth factor 1; ZEB1, zinc finger E‐box binding homeobox 1; miR‐183, microRNA‐183; RT‐qPCR, reverse transcription‐quantitative polymerase chain reaction.

### 2.9 Cell counting kit 8 (CCK‐8) assay

Prior to cell transfection, cells were pre‐incubated at 37°C with 5% CO_2_ in a 96‐well plate for 24 h. HTR‐8/SVneo cells were then transfected with oe‐IGF‐1 or oe‐NC after 0, 24, 48 and 78 h. Then, 10 μl of CCK‐8 solution (Beyotime Biotechnology Co., Ltd.) was added to each well and cultured for 4 h, and the optical density (OD) value at 450 nm was measured by a microplate reader.

### 2.10 In vitro angiogenesis experiment

HTR8/SVneo cells were transfected with si‐IGF‐1 or oe‐IGF‐1 plasmids, and the supernatant was collected and settled to a 96‐well plate containing 1.5 × 10^4^ human uterine microvascular endothelial cells (HUtMEC; PromoCell), which were coated with matrix gel. After 16 h, the HUtMEC branch was observed under an optical microscope and photographed. The total number of branch points and branch tubes was manually calculated from photomicrography.

### 2.11 Chromatin Immunoprecipitation (ChIP) assay

After treatment with 4% formaldehyde (final concentration 1%), the collected HTR‐8/SVneo cells were sonicated by ultrasonic, followed by the addition of the ZEB1 antibody and fully contacted with the miR‐183 promoter. Protein A Agarose/SaLmon Sperm DNA was added to the cells to allow binding to ZEB1 antibody‐PBX3‐OTX1 promoter complexes. The complexes were then precipitated and rinsed to remove the non‐specific binding and the enriched promoter complexes were obtained. RT‐qPCR was then conducted after the enriched promoter complexes were de‐crosslinked and purified.

### 2.12 Mouse model of PE construction

A total of 20 specific pathogen‐free grade CD‐1 mice (aged 6–8 weeks, weighted 25–30 g) were purchased from Vital River Laboratory Animal Technology Corp. (Beijing, China). Mice were raised with light time from 6 AM – 6 PM under fixed temperature of 2–26°C and relative humidity of 70% with free access to food and water. The feeding environment was disinfected by UV regularly, and ventilation was assured. The experiment was initiated after 1 week of feeding and adaptation. Female mice mated with fertile or vasectomized male mice at night, and successful mating was established by the appearance of vaginal plugs, indicating 12 h of pregnancy or pseudopregnancy. After the mice were euthanized, the placenta tissues were collected and immediately frozen and store at −80°C for future use.

The HIV‐I based self‐extinction lentiviral vector plasmid pLV‐EGFP was prepared as described previously ^24^, whereas the other shRNAs targeting target genes were prepared by substituting EGFP cDNA. The lentiviral packaged plasmids were transfected into 293 T cells. The virions were collected after 2 days post‐transfection, followed by centrifugation at 5000 *g* for 2 h and resuspension with potassium simplex optimized medium (KSOM) to adjust the concentration to 8 × 10^6^ TU/ml. Wild‐type CD1 female mice were ovulated by intraperitoneal injection of pregnant mare serum gonadotropin (PMSG; 5 units) and human chorionic gonadotropin (hCG; 5 units) 48 h later, then mated with wild‐type CD1 males. Embryos of blastocysts were harvested from mice at 4 d after mating, while the zona pellucida was removed in acidic Tyrode's solution (Sigma‐Aldrich,). In a 5 μl portion of medium containing the lentiviral vector, the blastocysts without the zona pellucida were cultured for 8 h. The transduced blastocysts were washed three times with KSOM and subsequently transplanted into pseudopregnant CD1 female mice or cultured for another 48 h, followed by examination under a confocal microscope.

If the systolic blood pressure and proteinuria on Day 18 of pregnancy were dramatically increased (110.12 ± 2.73 mmHg), the establishment of the mouse PE model was considered successful. The mice with successful modelling were divided into two groups: sh‐NC and sh‐IGF‐1, with 10 mice in each group.

### 2.13 Blood pressure measurement

Mice were tested for blood pressure on Days 6, 12 and 18 of pregnancy using a rat tail blood pressure meter (Kent International USA, Inc.,). The measurement was conducted at 8 AM. after mice were deprived of water for 2 h. When measuring blood pressure, the room temperature was kept at about 25°C, and the incubator was preheated so that body temperature remained at 37°C. Mice were placed in the incubator 10 min prior to the measurement. The test clamp was placed at the end of the mice tail and the blood pressure was measured after the heartbeat was stabilized. Each mouse was measured three times, and the average value was obtained.

### 2.14 Automated biochemical analysis

The urine of mice in each group was collected on the E18.5. The level of urine protein was determined by an automatic biochemical analysis. Urine was centrifuged at 1006.2 g for 10 min at 4°C, and the level of urine protein was determined using biprenore reagent (HPBIO‐R1253, Pengpai Technology company,). On the 21^st^ (final) day of pregnancy, the mice were not delivered, but the placenta was taken by caesarean section, part of which was used for extraction and total protein and RNA detection, whereas the other samples were fixed for subsequent experiments.

### 2.15 Haematoxylin–eosin (HE) staining

The placental tissues stored in 4% formalin were dehydrated twice in a series of increasing concentration of alcohol (75, 80, 90, 90 and 100%, respectively, each time for 1.5 h). Tissues were cleared t twice with xylene, for 8 min each time. After dehydration and transparentizing, tissues were embedded and sliced into section of 5–7 μm thickness (RM2016, Leica Co., Ltd.,). The slices were then baked at 55°C and dewaxed twice in xylene (5 min each time), conventionally dehydrated with a series of decreasing concentration of alcohol (100, 95, 85 and 75%, 3 min each time), stained with haematoxylin for 8 min and differentiated by hydrochloric acid alcohol for 5 s. The slices were returned to blue with 0.25% ammonia water for 1 min, followed by staining with eosin for 30 s, dehydrated twice at 85, 95 and 100% alcohol (2 min each time) and cleared twice in xylene (5 min each time). After staining, the slide was added with neutral gum and sealed with a clean coverslip. The pathological changes in the placenta were observed under the microscope (XSP‐2C, Shanghai Bingyu Optical Instrument Co., Ltd.,).

### 2.16 Statistical analysis

The SPSS 21.0 statistical software (IBM Corp,) was employed for statistical analysis. All measurement data were expressed as mean ± standard derivation. A comparison between the two groups was analysed by independent *t*‐test. Comparisons among multiple groups were analysed by one‐way analysis of variance (anova) and followed by Tukey's *post‐hoc* test. Comparison among groups at different time points was analysed by repeated‐measures anova and followed by Bonferroni's *post‐hoc* test. Enumeration data were expressed as a percentage or ratio. Correlations between data were analysed by Pearson correlation analysis. A *p* < 0.05 was considered as statistically significant.

## 3 RESULTS

### 3.1 IGF‐1 was poorly expressed in placenta tissues of preeclamptic patients

The clinical features of participants, including 18 women with PE and 18 in normal pregnancy (NP) are presented in Table 2. There was no significant difference in age between the subjects in the NP group and PE group, but systolic blood pressure, diastolic blood pressure and urine protein content increased remarkably in the PE group.

**TABLE 2:** Baseline characteristics of participants

Items	Normal pregnancy	PE	*p‐*value
Maternal age (years)	28.91 ± 3.36	29.64 ± 3.26	.513
Gestational age (weeks)	38.99 ± 1.18	35.91 ± 0.54	<.001
Systolic blood pressure (mmHg)	117.84 ± 10.67	149.92 ± 16.47	<.001
Diastolic blood pressure (mmHg)	75.09 ± 8.19	98.08 ± 12.09	<.001
Proteinuria (g/24 h)	–	3.09 ± 1.18	–
Infant birth‐weight (g)	3386.54 ± 368.58	2730.81 ± 280.88	<.001

**FIGURE 1:**
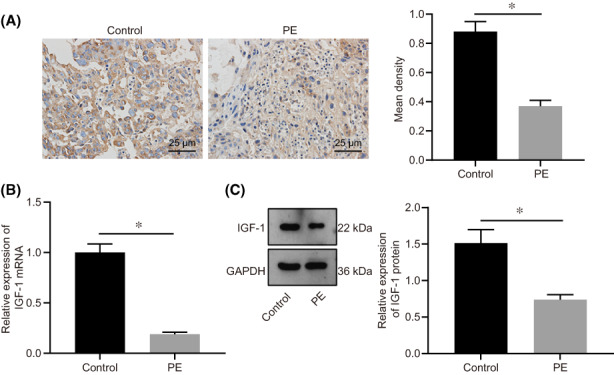
IGF‐1 was downregulated in the PE group. A, The expression of IGF‐1 in placental tissues of PE and NP determined by IHC (*n* = 18). B, IGF‐1 RNA expression in PE group measured by RT‐qPCR (*n* = 18). C, Protein expression of IGF‐1 in PE measured by Western blot analysis (*n* = 18), grey analysis was performed using Image J software. Data were all measurement data and expressed as mean ± standard deviation. The independent *t*‐test was employed for comparison between the two groups. * *p* < 0.05 compared with the NP group. IGF‐1, insulin‐like growth factor 1; PE, pre‐eclampsia; NP, normal pregnancy; RT‐qPCR, reverse transcription‐quantitative polymerase chain reaction; IHC, immunohistochemistry; n, number

The expression of IGF‐1 in placental tissues from the PE and NP groups was determined by IHC, showing that the expression of IGF‐1 in placental tissues of the PE group was lower than that in the NP group (Figure 1A). RNA and protein levels of IGF‐1 were determined by RT‐qPCR and Western blot analysis, showing that RNA and protein expressions of IGF‐1 were decreased dramatically in the PE group (Figure 1B, C). Hence, low expression of IGF‐1 was present in the placental tissues of PE.

### 3.2 IGF‐1 enhanced proliferation, invasion, and angiogenesis of trophoblast cells

**FIGURE 2:**
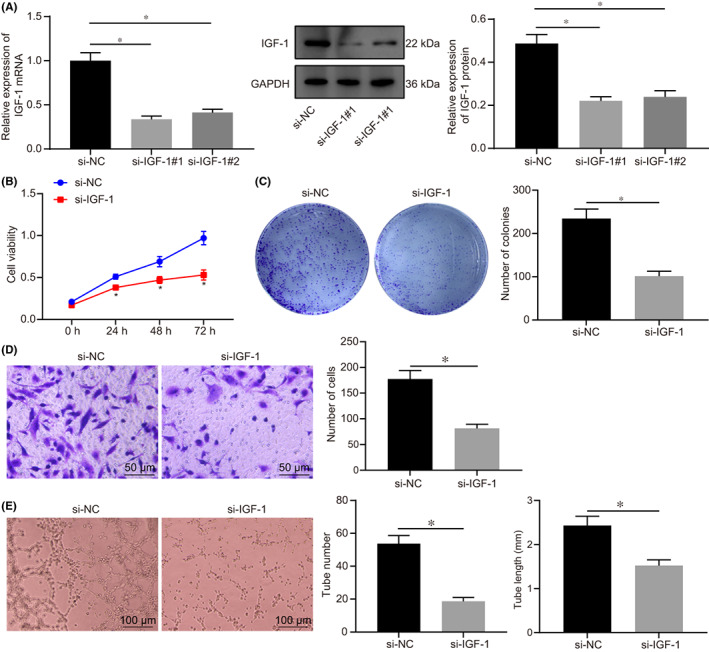
IGF‐1 promoted proliferation and invasion of HTR‐8/SVneo cells, as well as angiogenesis in vitro. A, The expression of IGF‐1 in HTR‐8/SVneo cells after transfected with si‐IGF‐1 (1) and si‐IGF‐1 (2) determined by RT‐qPCR. B, Cell viability at 0 h, 24 h, 48 h and 72 h after transfected with si‐IGF‐1 detected by CCK‐8. C, The clonality of cells after transfected with si‐IGF‐1 detected by colony formation assay. D, The invasive ability of cells after transfected with si‐IGF‐1 detected by Transwell assay, (scale bar = 50 μm). E, Detection of lumen formation detected by HUVECs angiogenesis assay and statistical analysis of lumen number and length (× 100). All experiments were repeated three times. Data were measurement data and expressed as mean ± standard deviation. The independent sample *t*‐test was used for comparison between two groups. Comparisons among multiple groups were analysed by one‐way anova and followed by Tukey's *post‐hoc* test. * *p* < 0.05 vs. that of cells transfected with si‐NC. CCK‐8, cell counting kit 8; anova, analysis of variance

Human chorionic trophoblast cells (HTR‐8/SVneo) were transfected with si‐IGF‐1 to explore the effect of IGF‐1 on the biological function of trophoblast cells. The efficiency of si‐IGF‐1 in HTR‐8/SVneo cells was assessed by RT‐qPCR. Results revealed that both si‐IGF‐1 (1) and si‐IGF‐1 (2) decreased the expression of IGF‐1 in HTR‐8/SVneo cells to about 33.6% (Figure 2A), while the decrease was more significant in the case si‐IGF‐1 (1). Hence, si‐IGF‐1 (1) was selected for follow‐up experiments. The effect of IGF‐1 on cell proliferation ability was detected by CCK‐8 and colony formation assays. Results demonstrated that, compared with cells transfected with si‐NC, the viability and clonality of cells were both suppressed after silencing of IGF‐1 (Figure 2B,C), indicating that silencing IGF‐1 could reduce proliferation of trophoblast cells.

In addition, the invasive ability of cells was detected by Transwell assay, which showed the invasive rate of cells was significantly reduced after transfection with si‐IGF‐1 (Figure 2D). HUVEC cells were tested for angiogenesis in vitro and the number and length of newly formed blood tubes were measured. Based on these observations, the number of angiogenesis was decreased and the length of newly formed blood tubes became shorter in cells transfected with si‐IGF‐1 compared with cells transfected with si‐NC, thus suggesting that angiogenesis was inhibited after downregulation of IGF‐1 (Figure 2E). According to these findings, knockdown of IGF‐1 could inhibit the proliferation and invasion of trophoblast cells, as well as angiogenesis in vitro.

### 3.3 IGF‐1 promoted the expression of ZEB1 through ERK/MAPK pathway

**FIGURE 3:**
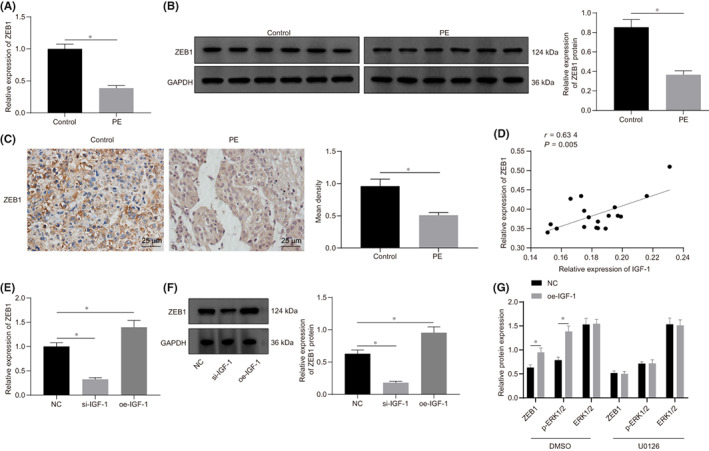
Expression of ZEB1 was elevated by IGF‐1 through the ERK/MAPK pathway. A, The RNA expression of ZEB1 in placental tissues of PE and NP detected by RT‐qPCR (*n* = 18). B, The protein expression of ZEB1 in placental tissues of PE and NP detected by Western blot analysis (*n* = 18), grey analysis was performed using Image J software. C, The expression of ZEB1 determined by IHC (*n* = 18) and the positive density was counted. D, ZEB1 was positively correlated with IGF‐1 expression (*n* = 18). E, The RNA expression of ZEB1 after HTR‐8/SVneo cells transfected with si‐IGF‐1 or oe‐IGF‐1 detected by RT‐qPCR. F, The protein expression of ZEB1 after HTR‐8/SVneo cells transfected with si‐IGF‐1 or oe‐IGF‐1 detected by Western blot analysis, the grey analysis was performed by Image J software. **G**, The expression of ZEB1, ERK1/2 and p‐ERK1/2 expression in the presence or absence of ERK/MAPK inhibitors in HTR‐8/SVneo cells transfected with oe‐IGF‐1 determined by Western blot analysis. All experiments were repeated three times. Data were measurement data and expressed as mean ± standard deviation. The independent sample *t*‐test was used for comparison between two groups. Comparisons among multiple groups were analysed by one‐way anova and followed by Tukey's *post‐hoc* test. * *p* < 0.05 vs. control group and NC group. The cell experiment was repeated three times. ZEB1, zinc finger E‐box binding homeobox 1; ERK1/2, extracellular signal‐regulated kinase 1/2; p‐ERK1/2, phosphorylated‐ERK1/2; NC, negative control

RT‐qPCR and Western blot analysis were employed to detect the expression of ZEB1 in the placental tissues of PE and NP. Results showed that the mRNA and protein levels of ZEB1 were notably decreased in PE tissues (Figure 3A,B). The result from IHC illustrated that the expression of ZEB1 was also decreased in PE tissues and was positively correlated with IGF‐1 expression (Figure 3C,D).

Next, HTR‐8/SVneo cells were transfected with si‐IGF‐1 and oe‐IGF‐1, respectively, and the expression of ZEB1 in HTR‐8/SVneo cells was determined by RT‐qPCR and Western blot analysis. Results demonstrated that the expression of ZEB1 was decreased by 32.4% in cells transfected with si‐IGF‐1, whereas the expression of ZEB1 was elevated when cells transfected with oe‐IGF‐1 (Figure 3E,F). According to results of Western blot analysis, the phosphorylation of ERK/MAPK was also regulated by IGF‐1 (Figure 3G). To verify whether IGF‐1 regulates the expression of ZEB1 via the ERK pathway, HTR‐8/SVneo cells were treated with ERK/MAPK inhibitor U0126 and transfected with oe‐IGF‐1. Results revealed that the expression of ZEB1 was inhibited by treatment with the ERK pathway inhibitor. Meanwhile, when the ERK pathway was inhibited, the overexpression of IGF‐1 could not effectively activate the expression of ZEB1, indicating that IGF‐1 regulated the expression of ZEB1 protein through the ERK/MAPK pathway in HTR‐8/SVneo cells.

### 3.4 The proliferation and invasion of trophoblast cells and angiogenesis were enhanced by ZEB1 through inhibiting miR‐183

**FIGURE 4:**
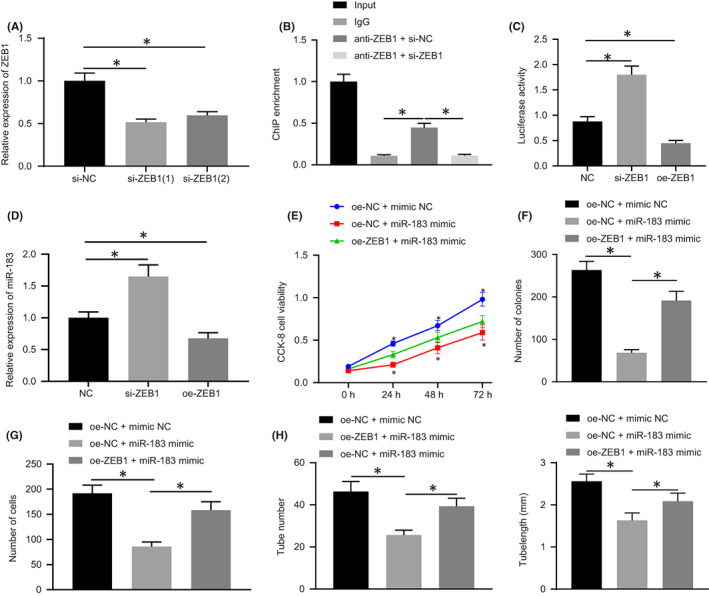
ZEB1 can promote the proliferation and invasion of HTR‐8/SVneo cells and angiogenesis in vitro by negatively regulating miR‐183. A, The expression of ZEB1 in HTR‐8/SVneo cells after transfection with si‐ZEB1 detected by RT‐qPCR. B, The binding between ZEB1 to miR‐183 promoter detected by ChIP‐PCR, * indicates comparison with the IgG group. C, The regulation of ZEB1 on miR‐183 detected by dual‐luciferase reporter gene assay. D, The expression of miR‐183 in HTR‐8/SVneo cells after transfection with si‐ZEB1 or oe‐ZEB1 measured by RT‐qPCR. E, Cell viability in cells transfected with miR‐183 mimic detected by CCK‐8 assay. F, The proliferation of HTR‐8/SVneo cells detected by colony formation assay. G, The invasion of HTR‐8/SVneo cells detected by Transwell assay. H, In vitro angiogenesis experiments. All experiments were repeated three times. Data were measurement data and expressed as mean ± standard deviation. Data from multiple groups were compared using one‐way anova and followed by Tukey's *post‐hoc* test. * *p* < 0.05 vs. si‐NC group, the NC group, the oe‐NC + mimic‐NC group and the oe‐ZEB1 + miR‐183 mimic group. The cell experiment was repeated three times. MiR‐183, microRNA‐183; IgG, immunoglobulin G; ChIP, Chromatin Immunoprecipitation

The effect of ZEB1 knockdown is presented in Figure 4A. Results from the ChIP‐PCR assay revealed that ZEB1 was capable of binding to the miR‐183 promoter and was reduced in cells transfected with si‐ZEB1 (Figure 4B). Dual‐luciferase reporter gene assay also confirmed that the transcription of miR‐183 was regulated by ZEB1 (Figure 4C). RT‐qPCR was conducted to investigate whether ZEB1 could affect the expression of miR‐183, and the results illustrated that the expression of miR‐183 was notably increased in HTR‐8/SVneo cells after being transfected with si‐ZEB1, whereas expression was significantly decreased after transfected with oe‐ZEB1 (Figure 4D). These results indicated that ZEB1 could act as a transcriptional repressor to regulate the expression of miR‐183.

Next, we examined whether miR‐183 affects the biology of trophoblast cells. HTR‐8/SVneo cells were transfected with miR‐183 mimic and cell proliferation was detected by CCK‐8 assay and colony formation assay. As shown in Figure 4E,F, cell proliferation was inhibited in cells transfected miR‐183 mimic. The invasive ability of cells was detected by Transwell assay, which showed that the invasive rate of HTR‐8/SVneo cells was dramatically decreased after transfection of miR‐183 mimic (Figure 4G). In vitro angiogenesis experiments also presented a significant reduction in angiogenic capacity after the transfection of miR‐183 mimic (Figure 4H). These phenomena were all rescued after transfection of ZEB1. Taken together, these results suggest that miR‐183 could inhibit HTR‐8/SVneo cell proliferation, invasion and angiogenesis, and that miR‐183 expression was regulated by ZEB1, which may affect the early development of the placenta.

### 3.5 IGF‐1 downregulation inhibited proliferation and invasion of trophoblast cells and angiogenesis in vitro could be reversed by silencing of miR‐183

**FIGURE 5:**
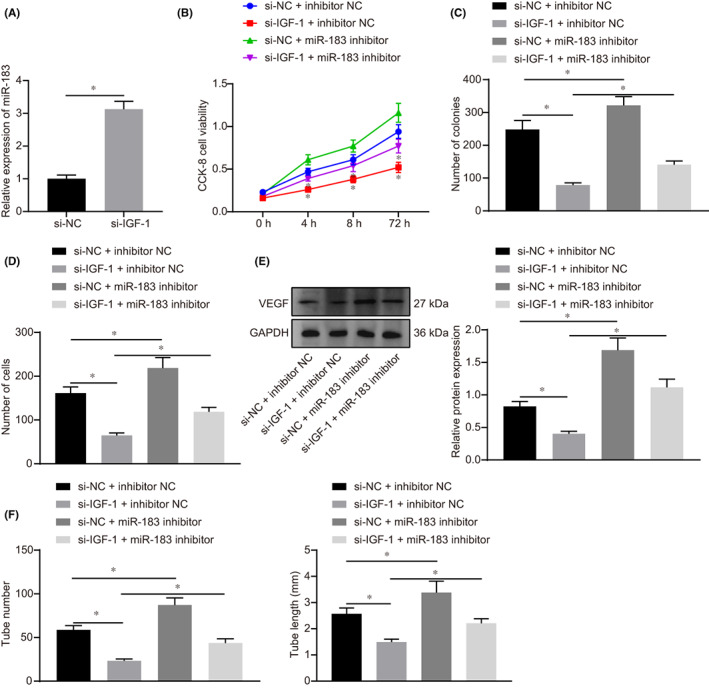
Silencing of miR‐183 reversed the suppression of proliferation and invasion of HTR‐8/SVneo cells and angiogenesis in vitro induced by IGF‐1 downregulation. A, The expression of miR‐183 after transfection of si‐IGF‐1 measured by RT‐qPCR. B, The viability of HTR‐8/SVneo cells measured by CCK‐8 assay after transfection of si‐IGF‐1 or miR‐183 inhibitor. C, The proliferation of HTR‐8/SVneo cells detected by colony formation experiments. D, The invasion of HTR‐8/SVneo cells detected by Transwell assay. E, The expression of VEGF measured by Western blot analysis. F, Detection of lumen formation detected by in vitro angiogenesis assay. All experiments were repeated three times. Data were measurement data and expressed as mean ± standard deviation. The independent sample *t*‐test was used for comparison between two groups. Comparisons among multiple groups were analysed by one‐way anova and followed by Tukey's *post‐hoc* test. * *p* < 0.05 vs. the si‐NC group, si‐NC + inhibitor‐NC and si‐IGF‐1 + inhibitor‐NC group. The cell experiment was repeated three times

We further examined whether the expression of miR‐183 was regulated by IGF‐1. Thus, HTR‐8/SVneo cells were transfected with si‐IGF‐1 and the cellular expression of miR‐183 was detected by RT‐qPCR. As expected, the expression of miR‐183 was notably increased in cells transfected with si‐IGF‐1 (Figure 5A). Next, HTR‐8/SVneo cells were transfected with a miR‐183 inhibitor to inhibit miR‐183, and CCK‐8 and colony formation assays were performed to detect cell proliferation. Results demonstrated that inhibition of proliferation caused by si‐IGF‐1 can be restored by miR‐183 (Figure 5B, C). Besides, the downregulation of miR‐183 also restored the reduced invasive ability of HTR‐8/SVneo cells induced by si‐IGF‐1 (Figure 5D). The expression of vascular endothelial growth factor (VEGF) was then assessed by Western blot analysis. Results indicated decreased expression after IGF‐1 knockdown, whereas an increased VEGF expression was observed after the silencing of miR‐183 (Figure 5E). In vitro angiogenesis experiments also confirmed that inhibition of miR‐183 reversed the repressed angiogenesis caused by si‐IGF‐1 (Figure 5F). In summary, these findings suggest that IGF‐1 could regulate miR‐183 by regulating ZEB1 expression, which affected the biological functions of trophoblast cells, ultimately involving in the pathogenesis of PE.

### 3.6 Silencing of IGF‐1 could induce the pathogenesis of PE

**FIGURE 6:**
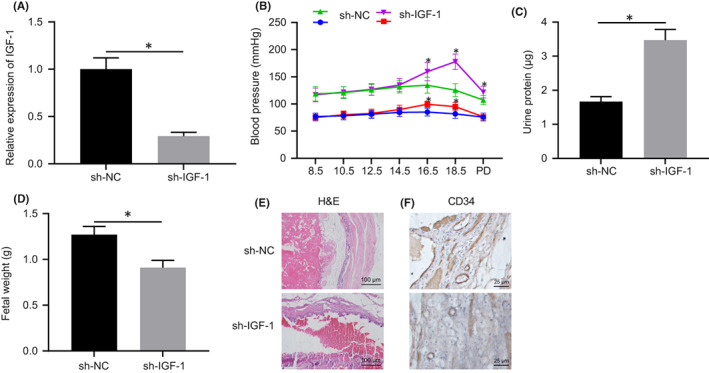
Silencing of IGF‐1 induces PE pathogenesis. A, The expression of IGF‐1 after transfection of sh‐IGF‐1 determined by RT‐qPCR. B, Systolic blood pressure and diastolic blood pressure in cells transfected with sh‐IGF‐1 group or sh‐NC at embryonic days E0, E8.5, E10.5, E12.5, E14.5, E16.5 and E18.5. C, Urine protein content in cells transfected with sh‐IGF‐1 of mice at E18.5. D, Weight of mice embryo. E, The structure of the placenta observed by HE staining (20 ×). F, The structure of the placenta observed by CD34 staining (20 ×). Data were measurement data and expressed as mean ± standard deviation. An independent *t*‐test was used for comparison between two groups. * *p* < 0.05 vs. that of cells transfected with sh‐NC. The cell experiment was repeated three times. HE, haematoxylin–eosin

To investigate the function of IGF‐1 in mice, we established a lentiviral‐mediated placental‐specific knockdown model and confirmed the model establishment by RT‐qPCR (Figure 6A). To determine whether sh‐IGF‐1 induces PE in mice, we measured blood pressure on embryonic days E0, E8.5, E10.5, E12.5, E14.5, E16.5 and E18.5. Results showed that systolic and diastolic blood pressure in the sh‐IGF‐1 group increased significantly on E16.5, and continued to increase during the rest of the pregnancy (*p* < 0.05, Figure 6B). Notably, the blood pressure returned to normal after delivery, which is similar to the phenomenon of human postpartum recovery. The urine protein levels measured at E18.5 were remarkably higher in the sh‐IGF‐1 group than in the NC group (Figure 6C). These findings indicated that sh‐IGF‐1 could induce a human‐like PE model, in which mouse body weight was significantly lower than the normal group (Figure 6D). Placental sections were subjected to HE‐staining, which showed that the structure of the placenta was disordered after sh‐IGF‐1 treatment (Figure 6E). Staining with CD34 antibodies also revealed a significant decrease in vascular density and branching, indicating that IGF‐1 plays an important role in mouse trophoblast cell proliferation and invasion, and the formation of blood vessels in the placenta (Figure 6F). In short, these results suggested that the silencing of IGF‐1 may implicate in the pathogenesis of PE.

## 4 DISCUSSION

Preeclampsia (PE) is a common complication of pregnancy, which threatens maternal and fetal health.^25^ Currently, placental delivery is the only radical solution for PE, whereas antihypertensive drugs were only effective for patients in a stable condition. However, eclampsia or lethal complications including stroke and pulmonary edema can occur if patients do not receive proper or timely treatments.^26^ The IGF system is thought to play a role in abnormal placentation, whereby IGF‐I activity is blocked and trophoblast invasion is reduced. Therefore, it has been suggested that pregnant women who later develop preeclampsia may have a lower concentration of IGF‐I in early pregnancy compared with other pregnant women.^27^ In the current study, the potential role of IGF‐1 in PE and the molecular mechanisms underlying the progression of PE were investigated. The experimental data confirmed the ameliorating effect of IGF‐1 on PE development, whereby IGF‐1 downregulates miR‐183 expression by increasing ZEB1 expression.

Initially, we observed low expression of IGF‐1 in the placenta tissues of patients with PE. To explore the effect of IGF‐1 on PE, trophoblasts were transfected with si‐IGF‐1, and the results presented that deregulation of IGF‐1 acts as a suppressor of cell proliferation, invasion and angiogenesis, thereby inducing PE in vitro. Consistent with our findings, other researchers also revealed the poor expression of IGF‐1 in women with PE compared with women in normal pregnancy.^28^, ^29^ Meanwhile, it has been reported that IGF‐1 was a significant regulator of proliferation, migration and invasion of trophoblast cells.^30^, ^31^ Moreover, it has also been validated that IGF‐1 plays a promoting role in the migration and invasion of extravillous trophoblast cells, which also involved in placental development and fetal growth by mediating placental functions.^32^ In addition, IGF bioavailability is modulated by specific binding proteins. Among these, insulin‐like growth factor binding protein‐1 (IGFBP‐1) is present in the decidua and at the decidua‐trophoblast interface, where it may act as a local modulator of IGF action on fetal growth.^33^ Previous research has showed that the abundance of IGFBP‐1 at the maternal‐fetal interface in severely preeclamptic pregnancies may participate in pathogenesis of the shallow placental invasion in PE, and that low circulating IGF‐1 and elevated IGFBP‐1 levels may contribute to restricted placental and therefore fetal growth.^34^ Determined secretion of IGFB1 in samples silenced for ZEB should be a matter for further investigation.

The present study further illustrated that IGF‐1 was able to upregulate ZEB1 expression through ERK/MAPK pathway. ZEB1 mediates a diverse array of processes including mesoderm‐derived cell differentiation, eye development, neural development and lymphopoiesis, as well as cell proliferation and senescence.^35^ In line with our findings, ZEB1 has been confirmed as a key transcriptional regulator of EMT, and its expression was elevated by IGF‐1 in prostate cancer cells.^36^ Moreover, it has been previously demonstrated that IGF‐1 could protect renal tubular epithelial cells from renal injuries via ERK/MAPK pathway activation.^37^ Besides, the ERK/MAPK pathway has also been demonstrated to be a crucial player in the appropriate development, differentiation and morphogenesis of the placenta.^38^ In the meantime, the nuclear localization of green fluorescent protein‐ZEB1 fusion clones has been identified to be disrupted by IGF‐1‐induced MEK/ERK phosphorylation.^35^ Interestingly, the present study revealed that the elevation of IGF‐1‐mediated ZEB1 resulted in reduced miR‐183 expression, thereby promoting the proliferation, invasion and angiogenesis of trophoblast cells, and thus alleviated the progression of PE in the mouse model. The downregulation of ZEB1 has been illustrated to be an inducer of PE by weakening the migration and invasive capacity of trophoblast cells.^39^ Moreover, the elevated miR‐20a levels in PE tissues have also been identified to inhibit the proliferation and invasion of trophoblast cells.^40^ Furthermore, miR‐15b proved to be an inhibitor of trophoblast cell invasion and angiogenesis of endothelial cells.^41^ Previous studies have also validated the relationship between aberrant expression of miR‐183 and the pathogenesis of PE. ^22^, ^42^ Till date, several studies have also certified the negative regulatory effect of ZEB1 on the expression of miR‐183.^43^, ^44^ In addition, miR‐183‐5p has been confirmed to be suppressed by ZEB1, which was associated with the development of cardiac hypertrophy and heart failure.^45^ Moreover, miR‐431 inhibited the migration and invasion of trophoblastic cells by targeting ZEB1, which might itself give rise to the onset of PE.^39^ IGF‐1 has also been confirmed to be repressed by miR‐30a‐3p in the placenta tissues of patients with PE, as well as repressed the invasive ability and ultimately elevated the apoptosis of trophoblast cells, which resulted in deteriorated PE.^46^ Taken together, ZEB1 can regulate negatively the expression of miR‐183 and positively regulate IGF‐1 expression, thus engaging in the development of PE development.

## 5 CONCLUSION

**FIGURE 7:**
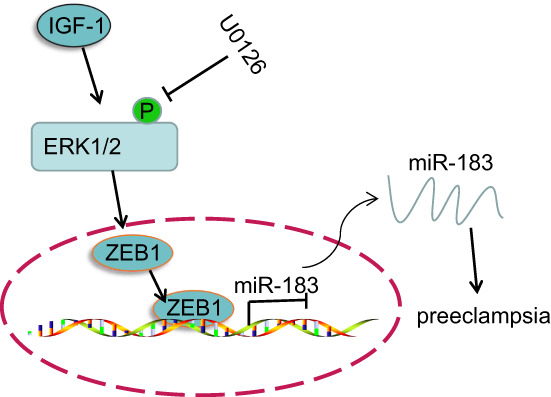
Role of the IGF‐1/ZEB1/miR‐183 axis in PE. IGF‐1 could inhibit PE development by attenuating miR‐183 expression through the elevation of ZEB1 expression

The results of the present study revealed that IGF‐1 protected against PE by decreasing miR‐183 expression through elevating ZEB1 expression (Figure 7). Results of this study thus support a model in which an IGF‐1‐mediated IGF‐1/ZEB1/miR‐183 axis modulates in the progression of PE, suggesting promising new therapeutic targets for PE. However, we expect that other available molecular mechanisms may also contribute to better clinical outcomes of PE management.

## 6 AUTHOR CONTRIBUTIONS

**Weisi Lai:** Conceptualization (lead); data curation (lead); formal analysis (lead); investigation (equal); methodology (equal); software (equal); validation (equal); writing – original draft (equal); writing – review and editing (equal). **Ling Yu:** Investigation (lead); project administration (equal); resources (lead); supervision (lead); validation (equal); visualization (lead); writing – original draft (equal); writing – review and editing (equal).

## 7 CONFLICTS OF INTERESTS

The authors declare that they have no conflicts of interests.

8

## Data Availability

All the data obtained and/or analyzed during the current study were available from the corresponding author on reasonable request.
